# Gene Co-expression Network Analysis of the Comparative Transcriptome Identifies Hub Genes Associated With Resistance to *Aspergillus flavus* L. in Cultivated Peanut (*Arachis hypogae*a L.)

**DOI:** 10.3389/fpls.2022.899177

**Published:** 2022-06-15

**Authors:** Mengjie Cui, Suoyi Han, Du Wang, Muhammad Salman Haider, Junjia Guo, Qi Zhao, Pei Du, Ziqi Sun, Feiyan Qi, Zheng Zheng, Bingyan Huang, Wenzhao Dong, Peiwu Li, Xinyou Zhang

**Affiliations:** ^1^College of Agriculture, Nanjing Agricultural University, Nanjing, China; ^2^The Shennong Laboratory, Henan Academy of Crops Molecular Breeding, Henan Academy of Agricultural Science, Zhengzhou, China; ^3^Key Laboratory of Oil Crops in Huang-Huai-Hai Plains, Ministry of Agriculture, Zhengzhou, China; ^4^Henan Provincial Key Laboratory for Oil Crop Improvement, Zhengzhou, China; ^5^National Centre for Plant Breeding, Xinxiang, China; ^6^Key Laboratory of Detection for Mycotoxins, Oil Crops Research Institute of the Chinese Academy of Agricultural Sciences, Ministry of Agriculture and Rural Affairs, Wuhan, China; ^7^Department of Horticulture, Ghazi University, Dera Ghazi Khan, Pakistan

**Keywords:** peanut, *Aspergillus flavus* L., resistance, transcriptome analysis, weighted gene co-expression network analysis (WGCNA)

## Abstract

Cultivated peanut (*Arachis hypogaea* L.), a cosmopolitan oil crop, is susceptible to a variety of pathogens, especially *Aspergillus flavus* L., which not only vastly reduce the quality of peanut products but also seriously threaten food safety for the contamination of aflatoxin. However, the key genes related to resistance to *Aspergillus flavus* L. in peanuts remain unclear. This study identifies hub genes positively associated with resistance to *A. flavus* in two genotypes by comparative transcriptome and weighted gene co-expression network analysis (WGCNA) method. Compared with susceptible genotype (Zhonghua 12, *S*), the rapid response to *A. flavus* and quick preparation for the translation of resistance-related genes in the resistant genotype (J-11, *R*) may be the drivers of its high resistance. WGCNA analysis revealed that 18 genes encoding pathogenesis-related proteins (PR10), 1-aminocyclopropane-1-carboxylate oxidase (ACO1), MAPK kinase, serine/threonine kinase (STK), pattern recognition receptors (PRRs), cytochrome P450, SNARE protein SYP121, pectinesterase, phosphatidylinositol transfer protein, and pentatricopeptide repeat (PPR) protein play major and active roles in peanut resistance to *A. flavus*. Collectively, this study provides new insight into resistance to *A. flavus* by employing WGCNA, and the identification of hub resistance-responsive genes may contribute to the development of resistant cultivars by molecular-assisted breeding.

## Introduction

As an important oilseed crop and a major source of vegetable oil and protein worldwide, peanut can be easily infected by *Aspergillus flavus* L. during drying, storage, and transportation processes (Nigam et al., 2009; Pandey et al., 2019; Soni et al., 2020), resulting in kernel rot and subsequent contamination of aflatoxins, which seriously threaten the safety of peanut products (Liang et al., 2006; Guo et al., 2008b; Passone et al., 2009; Ding et al., 2012). To date, breeding of cultivars with resistance to *A. flavus* has been widely accepted as the most cost-effective way to mitigate aflatoxin contamination, and the identification of hub resistance genes is recognized as a fundamental premise for breeding of resistance cultivars.

In general, pre- and post-harvest contamination are two major types caused by *A. flavus* (Guo et al., 2008a,b, 2011; Liao et al., 2009; Wang et al., 2013; Clevenger et al., 2016; Zhao et al., 2019; Soni et al., 2021). Two types of mechanism for host resistance to *A. flavus, in vitro* seed colonization and aflatoxin production, have been documented, which were further proved to be independently inherited (Liao et al., 2009; Nigam et al., 2009; Pandey et al., 2019; Soni et al., 2020), thus making it a great challenge for the researchers to elucidate resistance mechanism, breed resistant lines, and eventually control the disease in peanuts. Over the past two decades, studies have been conducted to characterize differentially expressed genes (DEGs) and main signaling pathways that were involved in resistance of *A. flavus*, but relatively limited information is available for hub genes associated with resistance to *A. flavus* stress, thus restricting the elucidation of *A. flavus*-resistance mechanism (Wang et al., 2016; Nayak et al., 2017; Walid et al., 2017; Korani et al., 2018). Recently, quantitative trait locus (QTLs) and single nucleotide polymorphisms (SNPs) related to peanut resistance to *A. flavus* have been reported (Liang et al., 2009; Pandey et al., 2014; Yu et al., 2019, 2020; Khan et al., 2020). However, no gene that responds to *A. flavus* in peanuts has been cloned by forward genetics. Meanwhile, studies on reverse genetics have demonstrated that pathogenesis-related proteins (PRs), such as PR10 (Luo et al., 2005; Guo et al., 2008a,b; Xie et al., 2013) and chitinase (Prasad et al., 2013), acted in the resistance to *Aspergillus flavus* L. infection. Although specific genes have been linked to peanut seed resistance, mining of hub resistance associated genes deserves further investigation.

Weighted gene co-expression network analysis (WGCNA), an approach that can be used to identify certain traits-related modules (Langfelder and Horvath, 2008; Menon, 2018), has been widely used in identification of hub resistance associated genes and clarification of molecular mechanisms of stresses in various plants (Hopper et al., 2016; Tan et al., 2017; Lin et al., 2019; Li et al., 2020; Liu et al., 2021; Yan et al., 2021). In this study, we aimed to systematically and comprehensively illustrate the resistance mechanism of two peanut genotypes that differ in their resistance to *A. flavus*, as well as identify hub genes positively associated with *A. flavus* resistance using WGCNA methods within RNA-seq analysis.

## Experimental Procedures

### Phenotypic Evaluation on the Resistance to *A. flavus* of Cultivated Peanut Genotypes

Experiments were conducted at the Henan Provincial Key Laboratory for Oil Crops Improvement, Henan Institute of Crop Molecular Breeding, Zhengzhou city, Henan, China. Highly toxigenic strain *A. favus* 3.4408 was cultured on dichloranglycerol-18 (DG-18) agar plates. After incubation for 7 days at 30°C, conidia were collected and suspended in sterile water containing 0.05% tween-80 with a concentration of 2 × 10^6^ CFU (spores/ml).

Approximately 200 healthy and mature kernels of each *R* (J-11) and *S* (Zhonghua 12) genotypes were collected for the experiment. Samples were collected at 0 (T0), 24 (T1), 48 (T2), 72 (T3), 120 (T5), and 168 h (T7) after inoculation from the infected samples of *R* and *S* genotypes. In total, 36 samples (2 genotypes × 6 stages × 3 replicates) were analyzed. At each time interval (T0, T1, T2, T3, T5, T7), 10–12 seeds were frozen in liquid nitrogen for RNA isolation and subsequently RNA-seq. Three seeds were immediately fixed by electron microscopy fixative and scanned in Wuhan Sevicebio Biological Technology, Wuhan city, Hubei, China. Seven days after inoculation, the infection index was scored according to the previously described method (Khan et al., 2020). Seeds were then autoclaved at 121°C for 30 min, and dried at 110°C for 3 h for aflatoxin B_1_ quantification by high-performance liquid chromatography (HPLC) method (Ma et al., 2013).

### RNA-Sequencing and Data Analysis

A total of 36 cDNA libraries were constructed using NEBNext Ultra RNA Library Prep Kit (NEB, USA) following the instructions of the manufacturer and deep sequenced by GENE DENOVO (Guangzhou, China), using Illumina sequencing platform. Three independent biological replications were used, and each biological replication contained five samples.

Reads were aligned to the reference *Arachis hypogaea* L. genome (GCA_003086295.2, https://www.ncbi.nlm.nih.gov/assembly) using HISAT 2. 2.4 with “-RNA-strandness RF” and other parameters set as a default (Kim et al., 2015). RSEM software was used to calculate the abundance values of the transcript based on the fragments per kilobase of exon per million mapped reads (FPKM) (Dewey and Li, 2011). DEGs were analyzed using DESeq2 software (Love et al., 2014), with the estimated absolute log_2_ fold change (FC) > 2 and false discovery rate (FDR) < 0.01. Gene Ontology (GO) and Kyoto Encyclopedia of Genes and Genomes (KEGG) enrichment analysis of DEGs were also carried out to identify *A. flavus* stress-related genes involved in the key biological processes and metabolic pathways associated with *A. flavus* response. Mapping of the top 30 significant enriched ones (p < 0.05) was generated on the online website (https://www.omicshare.com/tools/) by GENE DENOVO (Guangzhou, China), as described in the study by Li et al. (2021).

### Data Integration and Network Construction

Co-expression networks were constructed by the *R* package WGCNA (Langfelder and Horvath, 2008). Co-expression transcript was clustered into 16 modules after filtering the genes of which FPKM < 1 in more than half of the samples. CYTOSCAPE (v3.7.1) was then used to visualize the networks of genes within module and to present biological interaction of core genes (Shannon et al., 2003).

### RT-qPCR Analysis

Twelve genes were randomly selected for validating the repeatability and authenticity of gene expression patterns by RT-qPCR, as described previously (Guimaraes et al., 2012; Yin et al., 2013; Chen et al., 2014; Li et al., 2014; Cui et al., 2021). The alcohol dehydrogenase class III (*AhADH3, Arahy. VYWU26.2*) was selected as the internal reference (Supplementary Table S1) (Brand and Hovav, 2010).

## Results

### Cultivated Peanut Cultivars Exhibiting Higher Resistance (*R*) and Susceptibility (*S*) to *A. flavus*

**Figure 1:**
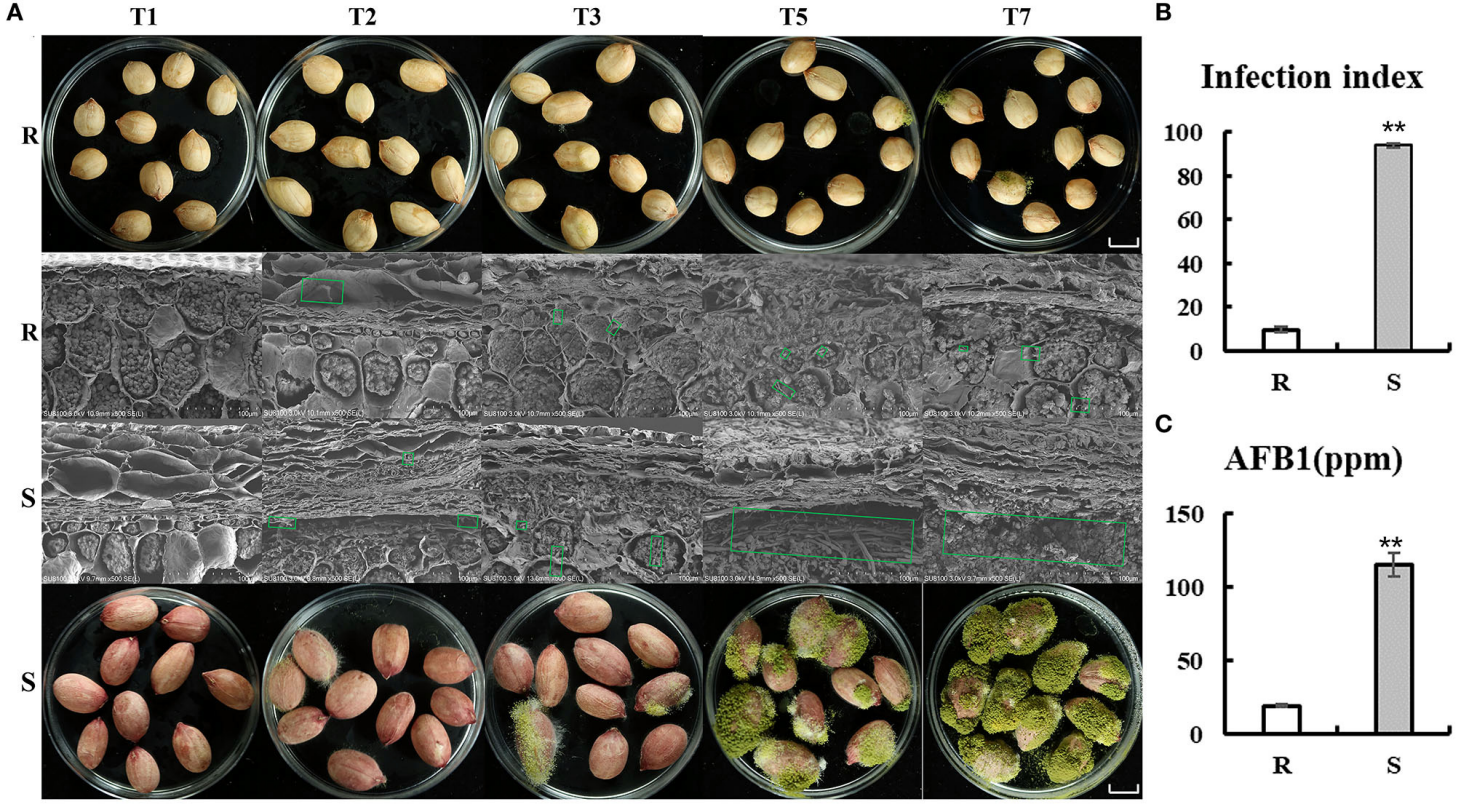
Comparison of *R* and *S* on the phenotypes of peanut seeds responding to *A. flavus*. **(A)** Mycelia growth on peanut seeds at different inoculation processes (T1, T2, T3, T5, and T7). Bars = 1 cm. **(B)** Infection index and aflatoxin B1 (AFB1) content **(C)** of the R and S genotypes after inoculation of *A. flavus* for 7 days. Green box shows mycelial of *Aspergillus flavus* L. Error bars indicated ± standard errors (SEs) of three independent biological replicates (*n* = 3). **P < 0.01 (*t*-test) compared with *R*.

Evaluation experiment of peanut resistance to *A. flavus* was performed by genotypes materials, which were grown in Yuanyang, China (N35°18′, E113°55′) (2020). Peanut accessions with higher resistance (J11, *R* cultivar) and susceptibility (Zhonghua 12, *S* cultivar) to *A. flavus* (Figure 1) as a comparative experimental material were selected. As seed coat of peanut, which is the outermost layer, may act as a physical barrier, the *A. flavus* infection process was observed by scanning electron microscopy (Figure 1A). Obviously, mycelial on the seeds of both of *R* and *S* penetrated the seed coat on the second day of infection (T2) and reached the cotyledons, where they acquired nutrients and produced aflatoxin (Figure 1A), and very little mycelial was observed in the *R* seed coat compared with *S* (Figure 1A). Furthermore, profuse mycelial growth and sporulation in genotype *S* was compared to genotype *R* on the third day after inoculation (T3) (Figure 1A). At T7, kernels of *S* were almost covered by green sporulation, and 115,391 (μg/kg) aflatoxin B_1_ was detected (Figures 1B,C). These results suggest that seed coat may be not the main reason for the phenotypic differences between *R* and *S*, but were more mechanism-based. The two genotypes are ideal candidates for studying the resistance mechanism of *A. flavus* stress on peanut seeds.

### Transcriptome Profiles of 36 RNA Libraries From Peanut Seeds Infected With *A. flavus* at Different Time Points

A total of 380,590,871,700 raw reads and 377,441,077,140 clean reads (clean ratio > 99.15 %) were obtained after filtering reads with low-quality. On average, 91.77% of reads could be mapped to the reference genome of peanut, except for *S*-T5 and *S*-T7, which contained more mycelium and spores in kernels, the average mapping genome ratio of which was only 23.12 and 17.27%, respectively (Supplementary Table S2). As the amount of sequencing (reads) increases, the number of genes detected for *S-T5* and *S-*T7 also increased and eventually tended to be saturated, implying the high accuracy of transcriptome sequencing results (Supplementary Figure S1). Pearson correlation coefficients among samples (Supplementary Figure S2) and principal component analyses (PCA) (Supplementary Figure S3) manifested a tremendous difference between samples at T0 and other time points (T1, T2, T3, T5, and T7), indicating the infection of *A. flavus* L. induced large transcription level perturbation in the peanut. The raw transcriptome reads were submitted to the NCBI Sequence Read Archive (SRA) database under accession: PRJNA825125.

### Identification of Differentially Expressed Genes

**Figure 2:**
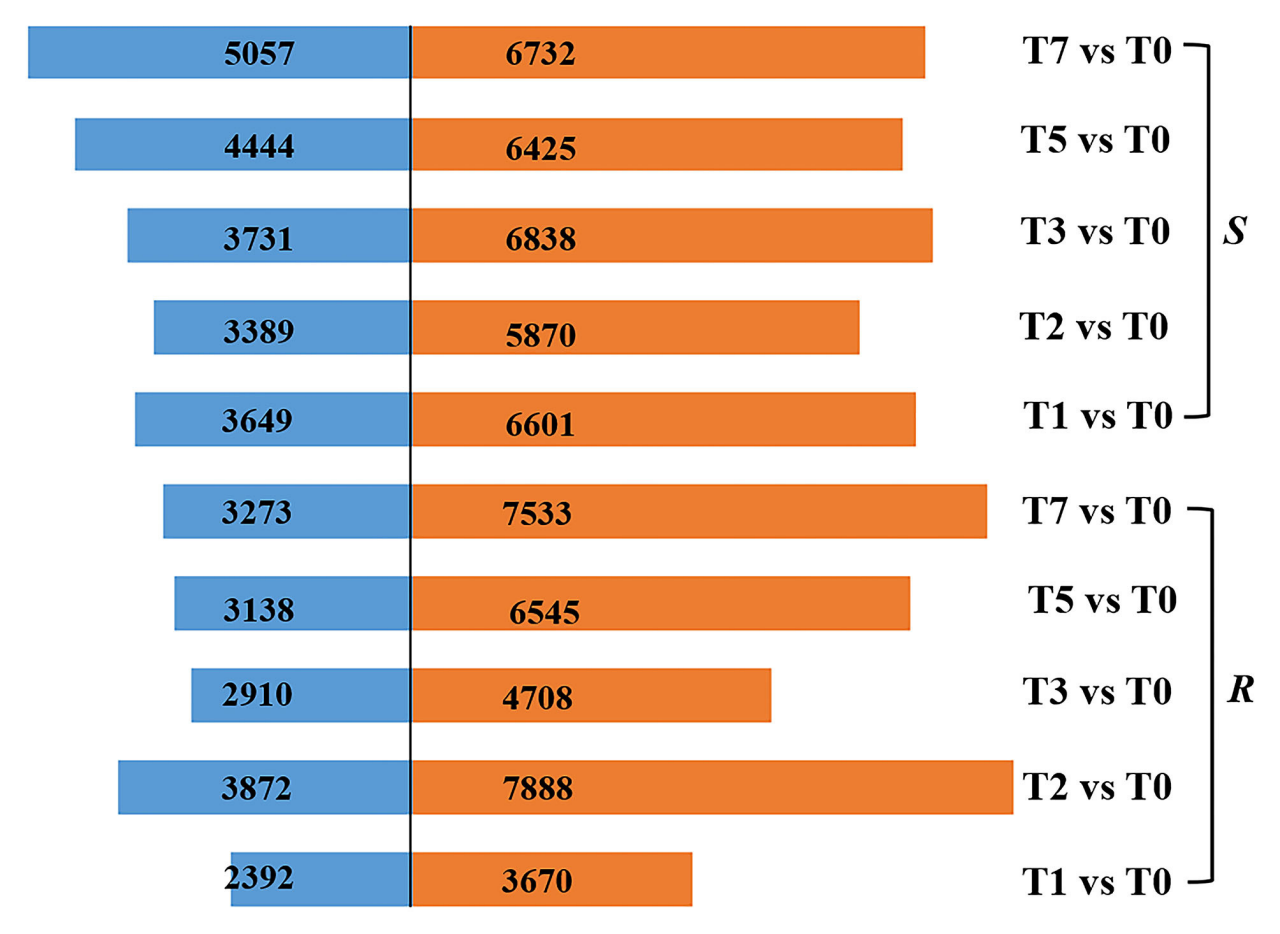
DEGs in *R* and *S* at five time points post-inoculation (T1, T2, T3, T5, and T7) compared with T0.

According to the criteria of |log_2_fold change| > 2 and FDR < 0.01, a total of 3,670, 7,888, 4,708, 6,545, and 7,533, respectively, upregulated DEGs and 2,392, 3,872, 2,910, 3,138, and 3,273 downregulated DEGs in *R* were screened, while 6,601, 5,870, 6,838, 6,425, and 6,732 DEGs were significantly upregulated and 3,649, 3,389, 3,731, 4,444, and 5,057 DEGs downregulated in *S* at T1, T2, T3, T5, and T7 after infection, respectively (Figure 2). It followed that DEGs (6,062–10,806) in *R* were fewer than those of *S* (10,250–11,789) at the stage of T1, T3, T5, and T7. Whereas the number of DEGs (11,760) in *R* was significantly higher than that in *S* (9,259) at T2, which was the time when mycelial penetrated seed coat, implying that *R* developed a very strong and quick resistant response to *A. flavus* at T2, thus preventing the seed from being contaminated by aflatoxin (Figure 2).

### DEGs Upregulated Uniquely in *R* Compared With *S*

**Figure 3:**
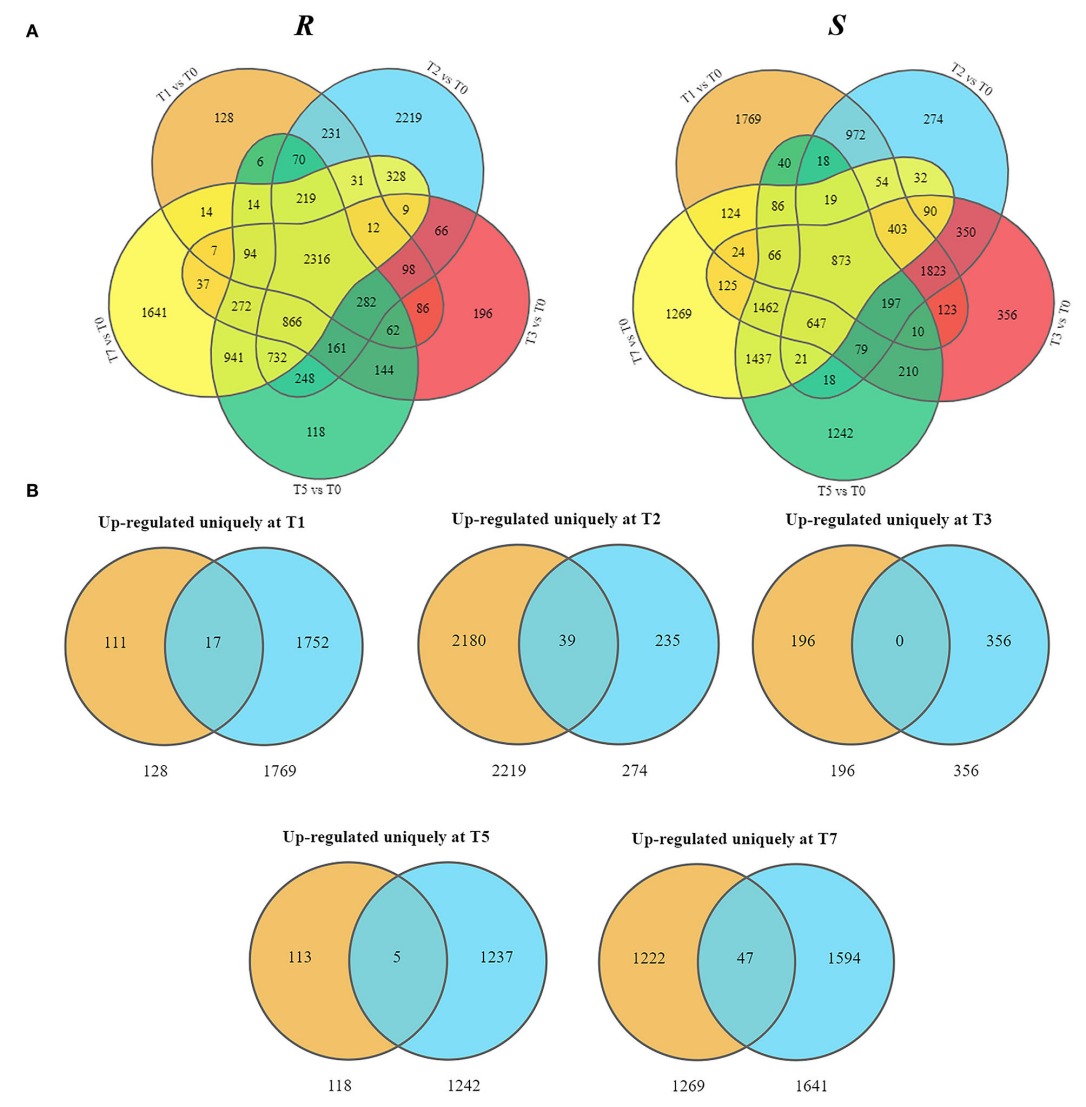
Presentation of upregulated DEGs in *R* and *S* at five time points post-inoculation (T1, T2, T3, T5, and T7). **(A)** Overlap analysis of upregulated DEGs. **(B)** Uniquely upregulated DEGs in *R* compared with *S*.

**Figure 4:**
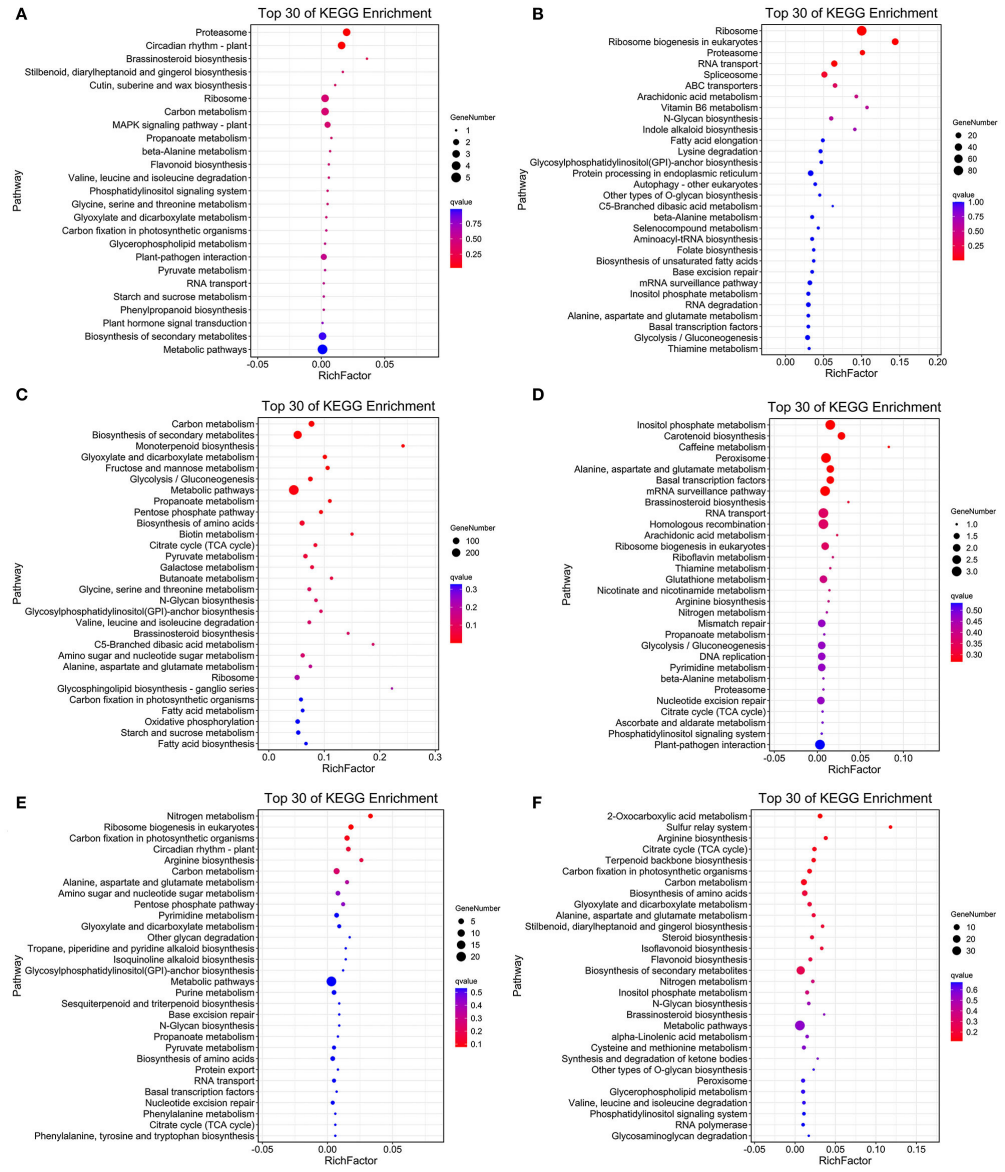
Top 30 of KEGG enrichment genes of uniquely upregulated DEGs in *R* and *S* at T1 **(A,B)**, T2 **(C,D)**, and T3 **(E,F)**.

As the upregulated genes may be positively responsible for resistance (Yan et al., 2021), we further conducted the overlap analysis at five-time points of *R* and *S*. The genes 128, 2,219, 196, 118, and 1,641 upregulated specifically DEGs in *R*, while 1,769, 274, 356, 1,242, and 1,269 in *S* were found at each time point (Figure 3A). For better identifying the critical time points of resistance in *R* and dissecting the resistance process, DEGs upregulated uniquely in *R* compared with *S* were explored further (Figure 3B). At T1, 111 DEGs were upregulated uniquely in *R*, while 1,752 DEGs were upregulated in *S*. GO analysis showed that the upregulated DEGs were enriched in “ribosome synthesis related process” and “response to endogenous stimulus” (Supplementary Figure S4). KEGG analysis indicated that these DEGs were enriched in “Brassinosteroid biosynthesis,” “MAPK signaling pathway—plant,” “Plant-pathogen interaction,” “Phenylpropanoid biosynthesis,” and “Plant hormone signal transduction” (Figure 4 and Supplementary Figure S5), which were reported to be main pathways of plants resistance to biotic stresses (Dixon and Paiva, 1995; Zhang and Klessig, 2001; Yan et al., 2018; Polturak and Osbourn, 2021). The same KEGG enrichment analysis revealed that the 1,752 DEGs, upregulated uniquely in *S*, were enriched in “Ribosome biogenesis in eukaryotes,” “Proteasome,” “RNA transport,” and “ABC transporters” (Figure 4 and Supplementary Figures S6, S7), but no resistance-associated pathways were significantly enriched uniquely in *S* at T1 (Supplementary Figures S6, S7), illustrating that *R* may respond more quickly than *S* and is prepared for resistance of infection at the level of transcription.

At T2, the unique upregulated DEGs (2,180) in genotype *R* were enriched in KEGG pathways such as “biosynthesis of secondary metabolites,” “Monoterpenoid biosynthesis,” “Fructose and mannose metabolism,” “Brassinosteroid biosynthesis,” and “Oxidative phosphorylation” (Figure 4 and Supplementary Figure S5), suggesting that genotype *R* may prepare for the translation and transportation of tolerance-related secondary metabolites at T2. Whereas in *S*, few pathways related to biotic resistance were enriched at T2 (Figure 4). Meanwhile, KEGG analysis of upregulated DEGs uniquely at T3, T5, and T7 DEGs in *R* was also enriched in disease resistance related pathways like “Plant-pathogen interaction,” “Autophagy—other eukaryotes,” “Phenylpropanoid biosynthesis,” “MAPK signaling pathway—plant,” “Ubiquitin mediated proteolysis,” and “SNARE interactions in vesicular transport” (Figure 4 and Supplementary Figure S5). It was observed that the pathways related to biotic resistance were significantly enriched in *S* at T3 (Figure 4 and Supplementary Figure S7), when profuse mycelial growth and sporulation started to appear (Figure 1). Taken together, it seems that the rapid responses of *A. flavus* in genotype *R* and activation of specific disease-related signaling pathways at T1 and T2 might lead to the high resistance.

### Co-Expression Network Analysis Identified Key Modules Correlated With Resistance to *A. flavus*

**Figure 5:**
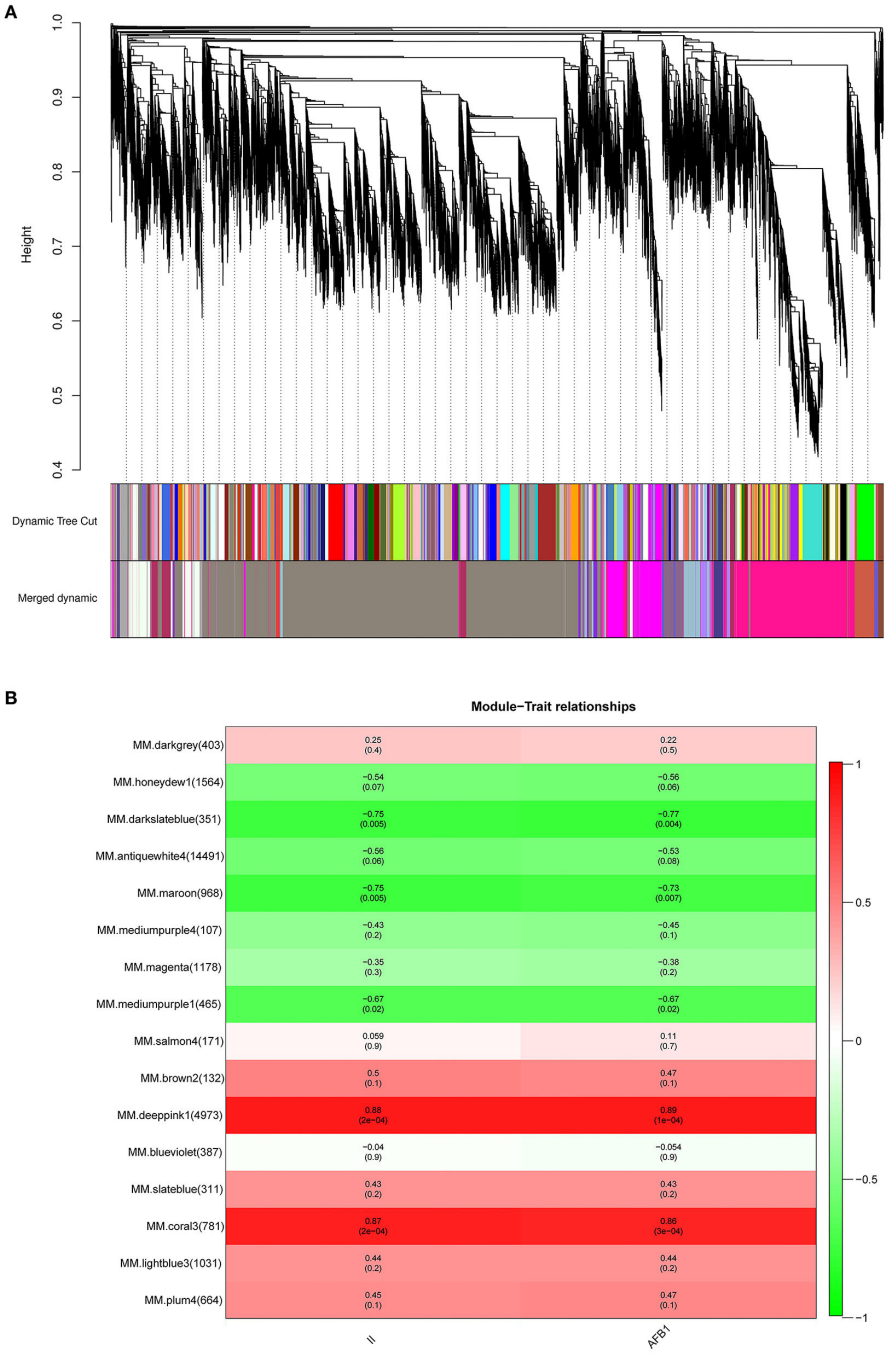
Co-expression network analysis of DEGs and module-trait relationship (MTRs) in response to *A. flavus*. **(A)** Cluster dendrogram of different genes in co-expression modules. **(B)** Relationships between modules (left) and traits (bottom). The numbers in brackets on the left show the number of genes in each module. Red and green represent positive and negative correlations, respectively. The darker colors indicate higher correlation coefficients. Numbers represent Pearson's correlation coefficients *r*^2^-values and the *P*-value for the correlation (in brackets).

To identify specific genes that were highly associated with resistance to *A. flavus*, WGCNA of 28,579 DEGs was carried out. We chose a power of β=12 based on the scale-free topology criterion to generate a hierarchical tree. All genes were assigned into 16 distinct modules (mergeCutHeight = 0.25) based on the similarity of their expression patterns (Figure 5A). The numbers of the genes in each module varied greatly ranging from 107 to 14,491 (Supplementary Table S3).

**Figure 6:**
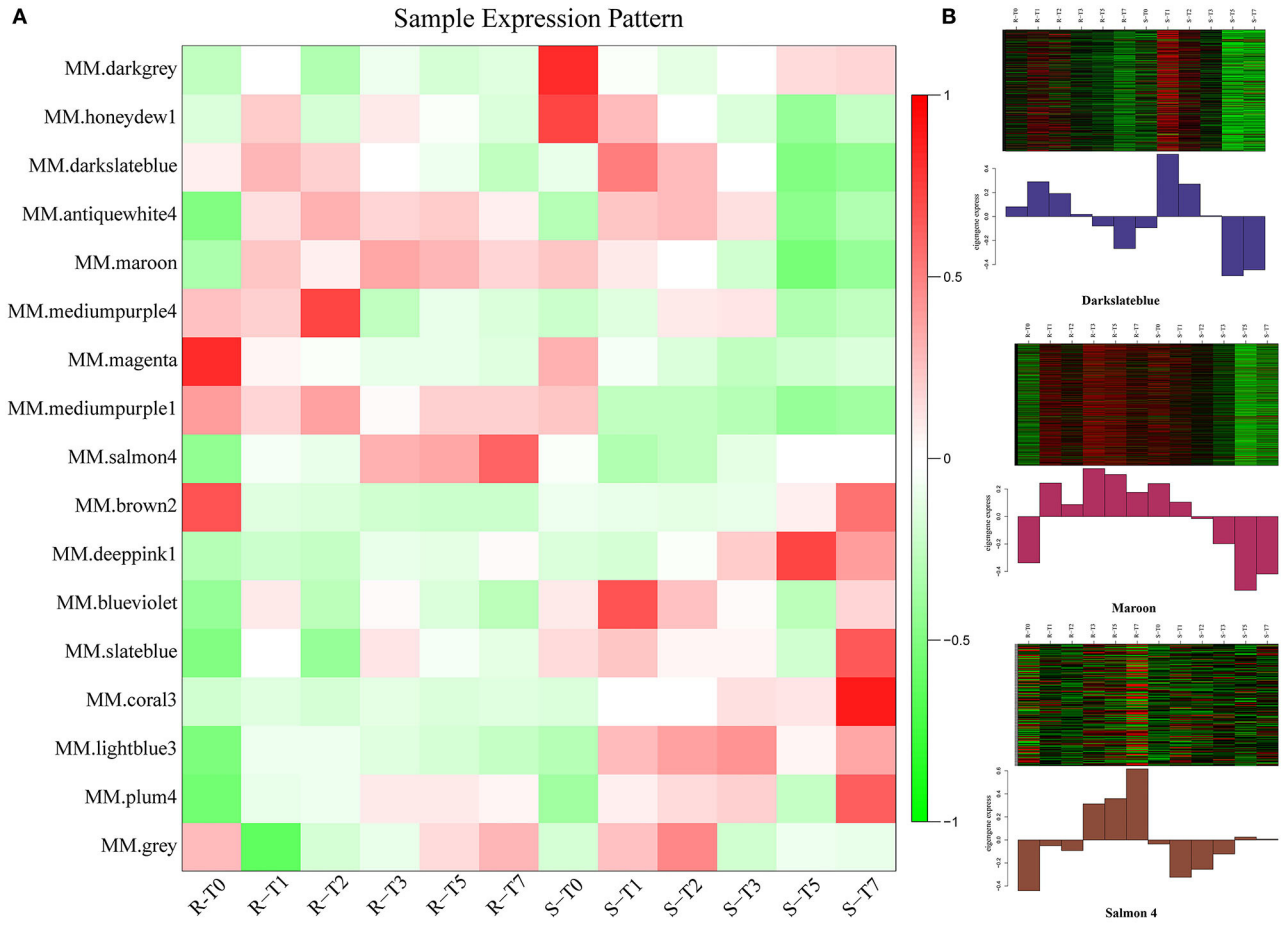
Expression patterns of genes in modules. **(A)** Heatmap of sample expression pattern. **(B)** Expression patterns in key modules at different time-points post-inoculation.

To characterize the key modules associated with *A. flavus* infection and aflatoxin production in peanut seeds, the module-trait relationships (MTRs) were analyzed subsequently (Figure 5B). Modules with MTR > 0.7 were selected as the key ones that were significantly associated with the growth and reproduction of *A. flavus* in seeds. Obviously, deeppink1 and coral3 were positively correlated with the infection index (II) and AFB1 content, whereas darkslateblue (*r*^2^ = −0.75/−0.77) and maroon (*r*^2^ = −0.75/−0.73) were negatively correlated with the corresponding traits, implying that DEGs in darkslateblue and maroon may act positively in inhibiting the growth of *A. flavus* and aflatoxin production in seeds. Subsequently, sample expression patterns were clustered and visualized by heatmap to clearly understand the expression of genes in modules at different time points after inoculation by *A. flavus*. As shown in Figure 6, expression level of genes in maroon and Salmon4 was increasing in R but with opposite trends in S with the extension of time after inoculation of *A. flavus*, and was higher in *R* than in *S* from T1 to T7, implying their positive roles in *A. flavus* resistance in *R*. Whereas the genes in darkslateblue expressed more strongly in *S* than in *R* from T1 to T3, suggesting its relatively low correlation with *A. flavus* defense. Heatmap of module-module relationship showed that maroon was significantly negatively correlated with deeppink1 (Supplementary Figure S8). In addition, plum 4 showed a significantly positive correlation with light-blue3 and coral3 but a negative correlation with magenta. And light-blue3 was significantly correlated with magenta and medium-purple1. Whereas there were no significant positive or negative correlation modules found with salmon4, implying the specificity of the module. All in all, it appears that genes in maroon and salmon4 may be the ones closely associated with resistance to *A. flavus* stress.

### GO and KEGG Enrichment Analysis of the Key Modules

GO and KEGG analysis of DEGs from the two key modules, salmon4 and maroon, was performed to clarify the specific functions of each module. GO analysis indicated that “response to biotic stimulus,” “defense response,” “response to stress,” “oxidoreductase activity,” and “protein kinase activity” were the most significantly enriched terms in Salman 4 (Supplementary Figure S9A). KEGG analysis revealed that “MAPK signaling pathway—plant,” “Plant-pathogen interaction,” “Phenylalanine, tyrosine, and tryptophan biosynthesis,” “Oxidative phosphorylation,” and “Plant hormone signal transduction” as the most significantly enriched metabolic pathways, indicating that genes in Salman 4 conferred the resistance to *A. flavus* by regulating “MAPK signaling pathway,” “Plant-pathogen interaction,” “Phenylalanine,” and “Oxidative phosphorylation,” and “Plant hormone signal transduction.” In Maroon, GO analysis identified “zinc ion binding,” “pyrophosphatase activity,” “hydrolase activity,” “cellular response to DNA damage stimulus,” “intracellular transport” as the most significantly enriched categories, and KEGG analysis identified “mRNA surveillance pathway,” “Basal transcription factors,” “Base excision repair,” “Glycine, serine and threonine metabolism,” and “Ubiquitin mediated proteolysis” as the most significantly enriched metabolic pathways, which showed that genes in Maroon module contribute to resistance of *A. flavus* by regulating mRNA surveillance, intracellular transport, and ubiquitin-mediated proteolysis (Supplementary Figure S9B).

### Hub Genes Involved in Resistance to *A. flavus* Screened *via* WGCNA

**Figure 7:**
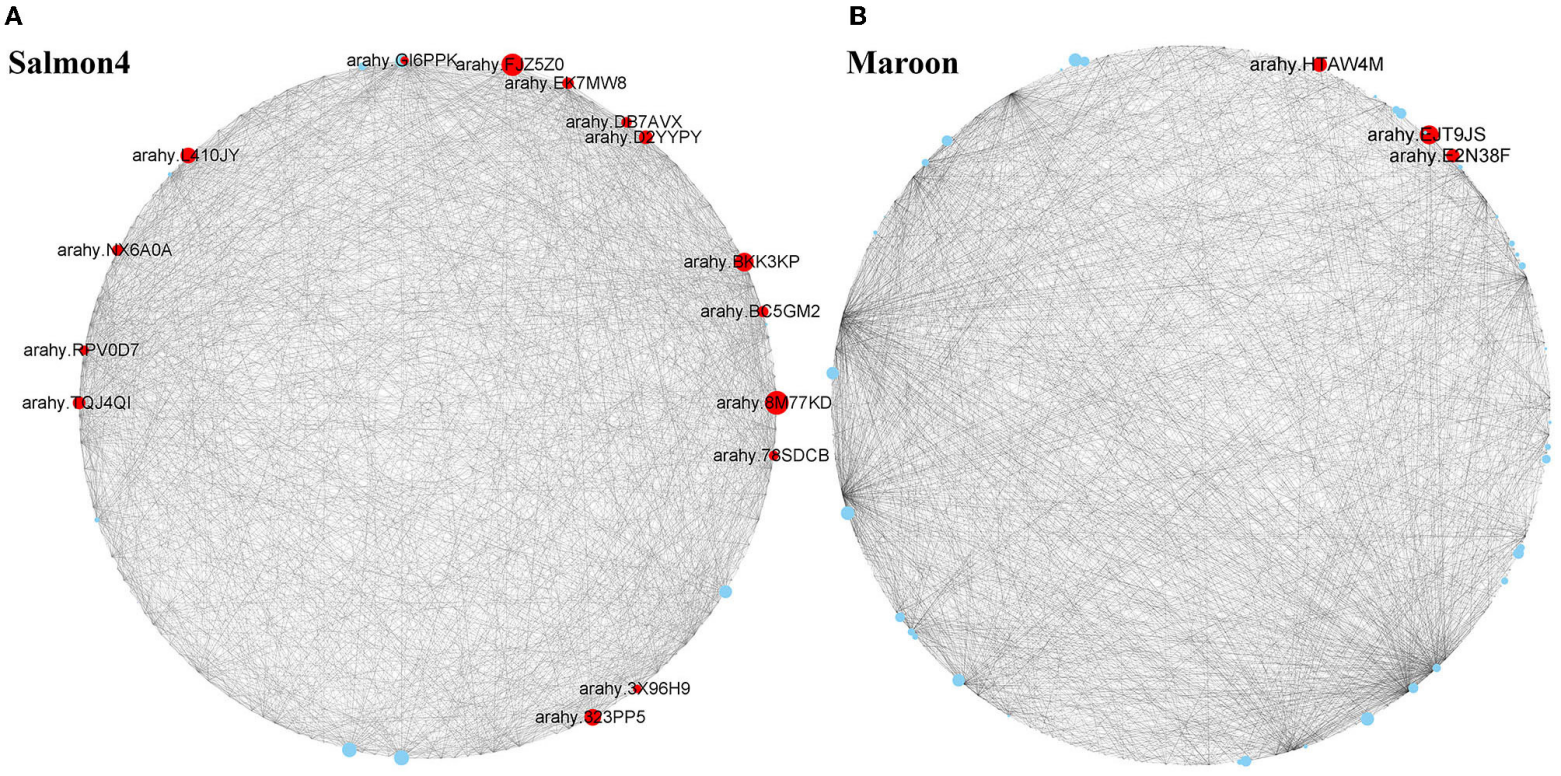
Hub genes identified in maroon and salmon4. Co-expressed network analysis of salmon4 module **(A)** and maroon module **(B)**. The size of the node circle is positively correlated with the number of genes it interacts with. Hub genes are shown as red nodes.

To identify the key genes associated with resistance to *A. flavus* in salmon4 and maroon, gene network analysis was conducted by CYTOSCAPE software (the first 2,000 edges) (Supplementary Tables S4, S5). After removing the unknown genes, the top 20 genes with the largest hubness with others were regarded as “hub genes” and shown as red nodes (Figure 7). Further information of the other genes in salmon4 and maroon is provided in Supplementary Tables S4, S5.

According to the function annotated in the reference genome, and annotation related to pathogen resistance (Ruperti et al., 2001; Assaad, 2004; Hollenstein et al., 2007; Jérme et al., 2007; Vierstra, 2009; Jayaprakash et al., 2019; Pandey et al., 2019; Zhao et al., 2019; Soni et al., 2020, 2021), 18 hub genes in the two modules were selected (Figure 7 and Table 1). In salmon4, six genes encoding pathogenesis-related 10 protein (PR10, *Arahy. 8M77KD, arahy. FJZ5Z0, arahy. BKK3KP, arahy. TQJ4QI, arahy. EK7MW8 and arahy. 3X96H9*) was characterized as members of Bet V 1 family protein, which were reported to function in degrading microbial nucleic acid for its ribonuclease activity (Bufe et al., 1996; Agarwal et al., 2013). *Arahy.78SDCB* and *Arahy.GI6PPK* are the ones encoding 1-aminocyclopropane-1-carboxylate oxidase (ACO1), which catalyze the formation of ethylene from 1-aminocyclopropane-1-carboxylic acid (ACC) and play key roles in ethylene signaling pathway (Johnson and Ecker, 1998; Bleecker and Kende, 2000; Lin et al., 2009). *Arahy. L410JY* and *arahy. BC5GM2* encode mitogen-activated protein kinase (MAPK protein) in MAPK cascades, which could sense the extracellular stimuli and conduct signaling transduction process (Zhang and Klessig, 2001). *arahy. D2YYPY* encodes serine/threonine kinase, which is one of the major types of disease resistance proteins (R) (Heierhorst et al., 2000). In addition, gene encoding pattern-recognition receptors (PRRs, *arahy. RPV0D7*), cytochrome P450 (*arahy. DB7AVX*), syntaxin of plants 121 (SYP121, *Arahy. NX6A0A*), and pectinesterase (*Arahy. 323PP5*) were also identified as hub genes with resistance to *A. flavus*. Three genes were identified in maroon. *Arahy. HTAW4M* and *arahy. E2N38F* encode phosphatidylinositol transfer family protein (PITPs), which is a class of proteins ubiquitous in eukaryotes that can promote the transfer of lipid molecules between intracellular membrane components, and participate in the signal transduction process of plant stresses (Phillips et al., 2006; Thole and Nielsen, 2008; Ghosh and Bankaitis, 2011). *Arahy. EJT9JS* is the one encodes pentatricopeptide repeat (PPR) superfamily protein that mainly regulates the expression of genes related to plant stress resistance through post-transcriptional modification of RNA (Small and Peeters, 2000; Ichinose and Sugita, 2017). All in all, 18 hub genes were identified as hub (key) genes that acted positively in resistance to *A. flavus* in peanuts and were classified into 10 categories (Table 1).

**Table 1:** Hub genes involved in resistance to *A. flavus*.

**Module**	**Categories**	**Hub genes**	**Connectivity**	**Functional annotation**
Salmon4	Bet v1 PR10	*arahy. 8M77KD*	32.33565012	Disease-resistance response protein
	Bet v1 PR10	*arahy. FJZ5Z0*	31.88090475	Disease-resistance response protein
	Bet v1 PR10	*arahy. BKK3KP*	30.8103282	Disease-resistance response protein
	Bet v1 PR10	*arahy. TQJ4QI*	28.93078647	Disease-resistance response protein
	Bet v1 PR10	*arahy. EK7MW8*	28.60674741	Disease-resistance response protein
	Bet v1 PR10	*arahy. 3X96H9*	27.58611499	Disease-resistance response protein
	ACO1	*arahy. 78SDCB*	28.00567838	1-aminocyclopropane-1-carboxylate oxidase
	ACO1	*arahy. GI6PPK*	27.50773409	1-aminocyclopropane-1-carboxylate oxidase
	MAPK kinase	*arahy. L410JY*	29.87068588	Protein kinase superfamily protein
	MAPK kinase	*arahy. BC5GM2*	28.42383101	Protein kinase superfamily protein
	STK protein	*arahy. D2YYPY*	29.23244119	Receptor serine/threonine kinase
	PRRs	*arahy. RPV0D7*	27.91178562	Receptor kinase 1
	Cytochrome P450	*arahy. DB7AVX*	28.22775289	Cytochrome P450, family 711
	SNARE protein SYP121	*arahy. NX6A0A*	28.37592007	Syntaxin of plants 121
	Pectinesterase inhibitor	*arahy. 323PP5*	30.13030689	Pectinesterase/pectinesterase inhibitor 17-like
Maroon	PITPs	*arahy. HTAW4M*	386.6120154	Sec14p-like phosphatidylinositol transfer family protein
	PITPs	*arahy. E2N38F*	382.8503316	Sec14p-like phosphatidylinositol transfer family protein
	PPR	*arahy. EJT9JS*	397.1419628	Pentatricopeptide repeat (PPR) superfamily protein

### Quantitative Real-Time PCR Validation of RNA-Seq Data

**Figure 8:**
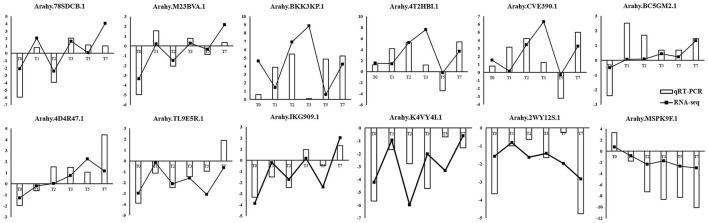
Validation of RNA-seq data by RT-qPCR. Y-axis showed the log_2_(R/S) between *R* and *S*. Positive value indicated upregulated in *R* and negative value indicated downregulated in *R*.

To validate the reliability of the RNA-seq data and differential expression level data, 12 genes were selected randomly from the DEGs to perform quantitative RT-qPCR (Figure 8). As shown in Figure 8, RT-qPCR detected the same expression tendency with the RNA-seq analysis. The validation experiments demonstrated that RNA-seq used in this study was highly reliable.

## Discussion

### Co-Expression Networks Were Constructed From Two Cultivated Peanuts With Differing Resistance to Aflatoxin Contamination Using the WGCNA Method

Plants are often subjected to a variety of biotic and abiotic stresses, of which *A. flavus* and subsequent aflatoxin contamination is an important example of biotic ones affecting food safety and peanut industry (Wild and Gong, 2010; Bryden, 2012; Sarma et al., 2017; Soni et al., 2020). Whereas the mechanism of resistance to *A. flavus* has not been elucidated and no hub resistance associated gene have been mined because of the lack of systematic screening method. In recent years, WGCNA have been recognized as an efficient method exploring hub genes related to certain traits in various plants (Amrine et al., 2015; Hopper et al., 2016; Kost et al., 2017; Tan et al., 2017; Lin et al., 2019; Wu et al., 2020).

In this study, we confirmed that *R* and *S* exhibit different responses to *A. flavus* stress based on RNA-seq data and morphological studies. The *R* and *S* cultivars exhibited reverse response phenotypes during the process of *A. flavus* puncturing kernel (Figure 1). Enrichment analysis of uniquely upregulated DEGs in *R* compared with *S* implied that high reactivity in *R* in T1 and T2 compared with other time points of inoculation. *R* genotype may respond more quickly than *S* genotype and prepare for the transcription and translation of resistance-related genes during the initial stage of infection. In addition, *S* had more DEGs than *R* regardless of the post-inoculation time except for T2, indicating that *S* needs to trigger more metabolic responsive processes and more genes to cope with the stress, which may owe to the lack of the coordination mechanism in *S* to adapt to stress. The responses shown by *R* and *S* during *A. flavus* infection process may elaborate differences for their resistance.

Moreover, modules closely associated with resistance to *A. flavus* were identified by WGCNA for the first time, and genes with the highest correlation with others were found and characterized as hub genes (Figure 7). Differential expression patterns of hub genes may lead to the obvious differences in phenotypes.

### *Aspergillus flavus* L. Perception and Recognition by PRRs in Peanut

Pattern recognition receptors (PRRs) play an essential role in pattern-triggered immunity (PTI) response (Shiu and Bleecker, 2001; Couto et al., 2016; Boutrot and Zipfel, 2017) for the detection of pathogen-associated molecular patterns (PAMPs) and initiation of immune signaling transduction (Jones and Dangl, 2006; Cao, 2016) by binding with various co-receptors, receptor-like kinases (RLK), and receptor-like cytoplasmic kinases (RLCK). And some studies also showed that PRRs, brassinosteroid insensitive 1-associated kinase 1 (BAK1) (Li et al., 2012; Zhang et al., 2020), fagellin sensitive 2 (FLS2) (Zhang et al., 2017), EF-Tu receptor (EFR) (Kim et al., 2011), chitin elicitor receptor kinase (CERK) (Petutschnig et al., 2010), and chitin elicitor-binding protein (CEBiP) (Dodds and Rathjen, 2010) were upregulated in the resistant lines of crops, thus acting positively in resistance to pathogens. In this study, one PRR gene, *arahy. RPV0D7*, was identified as hub gene that acted positively in the defense of *A. flavus*. It is inferred that peanuts can identify PAMPs of *A. flavus*, and induce PTI quickly when infected by *A. flavus*, and the rapid and active response of PRRs in *R* may be the major reason for the difference in the phenotypes between *R* and *S*.

### Serine/Threonine Kinase Disease Resistant (R) Genes

Serine/threonine kinase (STK) is one of the major types of disease resistance proteins (R), which are sorted into other four categories, namely, detoxifying enzymes, NB-LRR proteins, transmembrane receptor protein with leucine-rich repeat structure, and protein kinase with leucine-rich repeat structure (Pamela, 1997; Heierhorst et al., 2000). In previous research, Xa21 (Song et al., 1995), Pto (Martin et al., 1993), and Lr10 (Feuillet et al., 1997) were reported to belong to the STK group and were considered as candidate genes to induce resistance against diseases. *Arahy.D2YYPY.1* from salmon 4 module was identified as R gene by WGCNA, suggesting that *R* genotype triggered stronger ETI responses than *S* at the initial stages of stress and conferred resistance against *A. flavus*.

### Pathogenesis-Related Proteins

Previous studies have shown that PR family genes, especially *PR10* genes, can enhance the resistance against both biotic and abiotic stresses in plants (Wan et al., 2008; Gupta et al., 2013), such as sugarcane (Peng et al., 2017), plum (El-Kereamy et al., 2008), and maize (Xie et al., 2010). In this study, six *PR10* genes were identified as hub genes by WGCNA analysis from Salmon 4 and showed continuous upregulation from T1 to T7 in *R* vs. *S* genotype, signifying that *PR10* members were closely related to peanut seed resistance to *A. flavus* stress (Figure 7). Based on our data, it is speculated that the upregulation of PR10 in *R* genotype from T1 to T7 may contribute to inhibiting the growth of *A. flavus* in the seed.

### The Other Key Genes Involved in Resistance to *A. flavus*

It is reported that phosphorylation cascades, containing calcium-dependent protein kinases (CDPKs) and mitogen-activated protein kinase (MAPK), play a major role in PRR-derived signals transmission (Young et al., 2010; Saijo et al., 2018). Correspondingly, in the process of peanut resistance to *A. flavus*, there were two hub MAPK genes (*arahy. L410JY, arahy. BC5GM2*) detected, which were upregulated in *R* compared with *S* (Table 1). Another important hub resistance-associated gene set identified was cytochrome P450 (*arahy. DB7AVX*). Gunupuru et al. (2018) reported that *TaCYP72A*, one of the members of cytochrome P450 genes in wheat, indirectly improved the early resistance of wheat to *F. graminearum*. Likewise, *GbCYP86A1-1* gene in *Gossypium barbadense* was conformed to confer resistance against *Verticillium dahlia* by significantly increasing the expression of disease-associated genes (Wang et al., 2020). In addition, as a key enzyme in ethylene biosynthetic pathway, 1-aminocyclopropane-1-carboxylic acid (ACC) is widely used as a proxy for ethylene, given that nearly all plant tissues readily convert it into ethylene (Yu et al., 1979). Helliwell et al. (2013) reported that overexpression of the ACC synthase gene (*OsACS2*) significantly increases the endogenous ethylene content of rice and enhances rice sheath blight resistance. Two ACC genes (*Arahy.78SDCB.1* and *Arahy.GI6PPK.1*) were characterized as hub genes associated with *A. flavus* stress in peanut, suggesting that ethylene-mediated resistance responses may play an unignored role in resistance to *A. flavus*. Another hub gene identified in salmon4 was *arahy. 323PP5* encoding pectinesterase or pectinesterase inhibitor. It is reported that pectin is the main component of plant cell wall, exists in the intercellular layer and the middle layer, and affects the rheology and adhesion properties of cells. Overexpression of *AtPMEI2 and AtPMEI3* enhanced the resistance to *Botrytis cinerea* (Lionetti et al., 2017). Yang et al. (2019) reported that the overexpression of poplar *PtoPME35* in *Arabidopsis thaliana* controls stomatal opening and closing of leaf under mannitol stress, thereby regulating plant stress resistance (Yang et al., 2019). In *Arabidopsis*, SYP121, also known as SYNTAXIN RELATED PROTEIN1/PENETRATION1 (PEN1), is one of the SNARE (soluble N-ethylmaleimide-sensitive factor attachment protein receptor) proteins and encodes syntaxin that has been shown to reside on the plasma membrane (Collins et al., 2003) and is more specifically involved in the polarized secretion events that give rise to papilla formation during fungi attack in *Arabidopsis* (Assaad, 2004). One hub gene encoding SYP121 (*Arahy.NX6A0A.1*) identified from Salmon 4 module by WGCNA analysis implied that a more severe papilla response exists in *R* compared with *S* genotype, thus eventually preventing the *A. flavus* infection in kernels.

In addition, two genes annotated as member of sec14p-like phosphatidylinositol transfer family (PITPS, *arahy. HTAW4M* and *arahy. E2N38F*) were identified by the WGCNA method from DEGs (Table 1). As one of the proteins responsive for the transferring of lipid molecules between intracellular membrane components, PITPs were reported to be involved in the signal transduction process of plant stresses (Thole and Nielsen, 2008). Therefore, *arahy. HTAW4M, arahy. E2N38F* may also be a core factor in the responsive process. PPR is a type of protein containing PPRs (Small and Peeters, 2000). As a trans-acting factor, PPRs mainly regulate the expression of genes related to plant growth and development through post-transcriptional modification of RNA (Ichinose and Sugita, 2017). Studies have shown that stresses usually cause severe damage to the structure and function of plant mitochondria, and PPR protein can regulate mRNA processing by editing and splicing mitochondrial RNA, and thus playing an indispensable role in the response of plants to stress (Umbach et al., 2005; Baldwin and Dombrowski, 2006; Ma et al., 2006; Yan et al., 2006; Baxter et al., 2007). *Arabidopsis AtPPR96* is involved in mediating oxidative stress responses (Liu et al., 2016), and overexpression of the *Arabidopsis* PPR gene *SOAR1* can enhance the tolerance to salt, drought, and chilling damage (Tan et al., 2014; Jiang et al., 2015; Xing et al., 2018). Similarly, the hub gene *arahy. EJT9JS* encoding PRR gene was upregulated in *R* relative to *S* genotype, implying the positive regulation in resistance to *A. flavus*.

**Figure 9:**
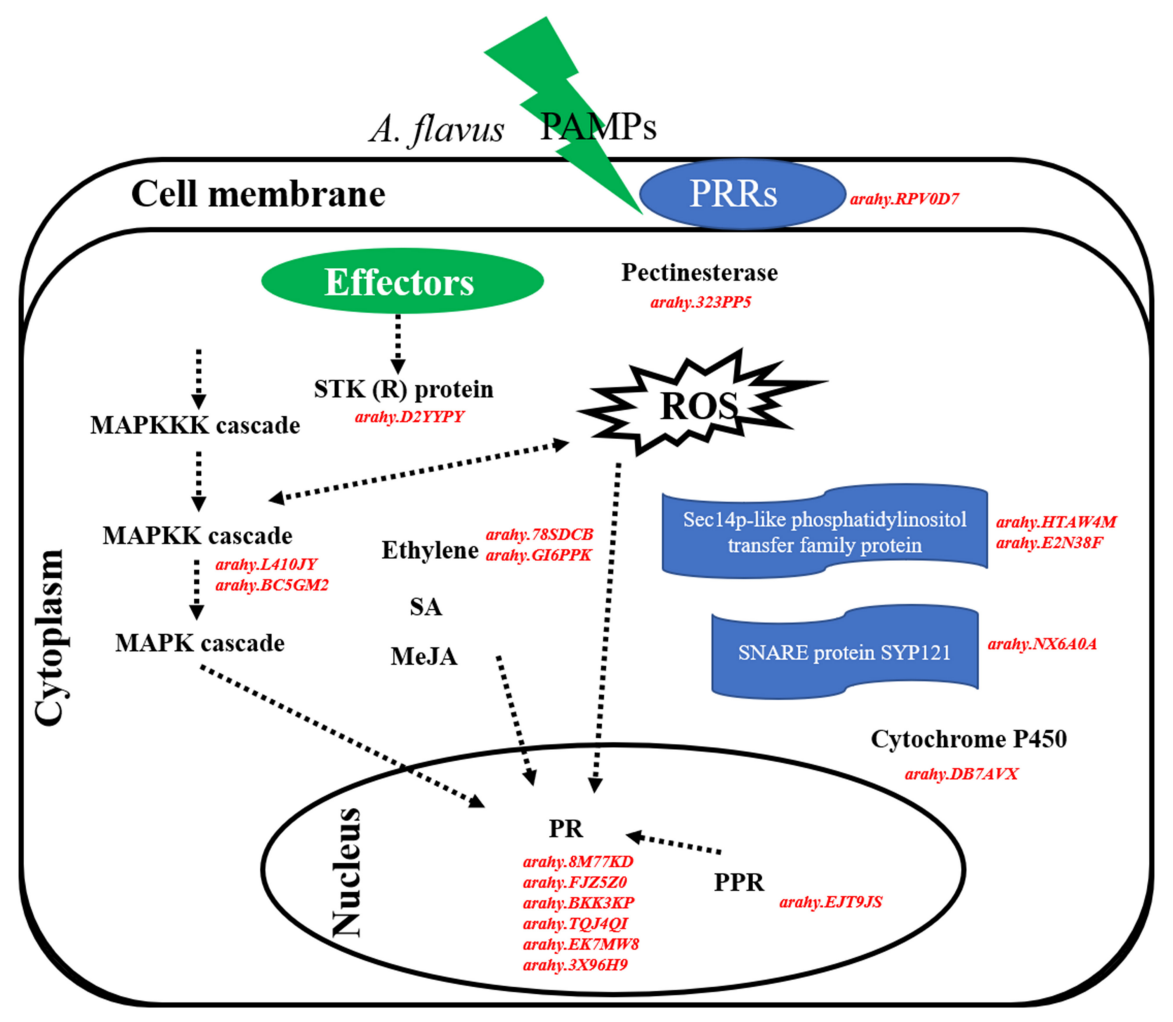
Hypothetical pathways and reactions present in response of peanut seeds to *A. flavus* at the gene expression level. The components represented in red color are hub genes resistance to *A. flavus* stress identified in our study. In brief, PAMPs of *A. flavus* combined with PRRs at the cell membrane and activate PTI response in peanut to limit the growth and reproduction of *A. flavus*. Subsequently, effectors of *A. flavus* were released into the cell and were recognized by R proteins (STK), thus triggering ETI response. In this defense responsive mechanism, PR10, pathogenesis-related proteins; ACO1, 1-aminocyclopropane-1-carboxylate oxidase; MAPK kinase, STK, serine/threonine kinase, PRRs, cytochrome P450, SNARE protein SYP121, pectinesterase, phosphatidylinositol transfer protein, and PPR protein are expressed during *A. flavus* infection process and play main role in the resistance mechanism.

Overall, 18 hub genes identified from salmon4 and maroon were the candidate genes in resistance to *A. flavus* stress. It is inferred that peanuts can identify PAMPs of *A. flavus*, and induce PTI and subsequently ETI when infected by *A. flavus* (Figure 9). The rapid and active response of PRRs, R genes, and other genes involved in a series of signaling pathways in *R* may be the major reason for the difference in the phenotypes between *R* and *S*. Certain regulatory function of hub genes will be further investigated in the future study.

## Conclusion

A total of 18 genes were identified, which might be associated with resistance to *A. flavus* in peanut. The upregulation of genes encoding pathogenesis-related proteins (PR10), 1-aminocyclopropane-1-carboxylate oxidase (ACO1), MAPK kinase, STK, PRRs, cytochrome P450, SNARE protein SYP121, pectinesterase, phosphatidylinositol transfer protein, and PPR protein involved in PTI and ETI response in *R* compared with *S* from T3 to T7 may be the cause of *R* showing resistance to *A. flavus*. Our study provides a new insight into future peanut breeding and development of *A. flavus* resistant peanut varieties to mitigate aflatoxin contamination for food safety and peanut industry.

## Data Availability Statement

The data presented in the study are deposited in the NCBI Sequence Read Archive (SRA) repository, accession number PRJNA825125.

## Author Contributions

MC, SH, and XZ conceived the study and research plans. MC, JG, and DW collected plant materials and performed the experiments. MC, QZ, and MH analyzed the data. PD, ZS, and FQ participated in handling figures and tables. MC, ZZ, and BH drafted the manuscript. WD, PL, and XZ revised the manuscript. All authors read and approved the final manuscript.

## Funding

This study was supported by the China Agriculture Research System (CARS-13), the Key Scientific and Technological Project of Henan Province (201300111000), and the Henan Provincial Agriculture Research System, China (S2012-5).

## Conflict of Interest

The authors declare that the research was conducted in the absence of any commercial or financial relationships that could be construed as a potential conflict of interest.

## Publisher's Note

All claims expressed in this article are solely those of the authors and do not necessarily represent those of their affiliated organizations, or those of the publisher, the editors and the reviewers. Any product that may be evaluated in this article, or claim that may be made by its manufacturer, is not guaranteed or endorsed by the publisher.

## Supplementary Material

Supplementary Figure S1 Distribution of sequencing saturation of *S*-T5 and *S*-T7.

Supplementary Figure S2 Pairwise Pearson's correlation coefficients of the sequencing data of 36 samples.

Supplementary Figure S3 Principal component analysis of the sequencing data of 36 samples.

Supplementary Figure S4 Top 30 GO term enriched functional categories of DEGs upregulated uniquely in *R* at T1 **(A)**, T2 **(B)**, T3 **(C)**, T5 **(D)**, and T7 **(E)**.

Supplementary Figure S5 Top 30 of KEGG enrichment of DEGs upregulated uniquely in *R* at T1 **(A)**, T2 **(B)**, T3 **(C)**, T5 **(D)**, and T7 **(E)**.

Supplementary Figure S6 Top 30 GO term enriched functional categories of DEGs upregulated uniquely in *S* at T1 **(A)**, T2 **(B)**, T3 **(C)**, T5 **(D)**, and T7 **(E)**.

Supplementary Figure S7 Top 30 of KEGG enrichment of DEGs up-regulated uniquely in *S* at T1 **(A)**, T2 **(B)**, T3 **(C)**, T5 **(D)**, and T7 **(E)**.

Supplementary Figure S8 Heat map of module-module relationship.

Supplementary Figure S9 Top 30 GO term enriched functional categories and top 30 KEGG enrichments of DEGs in salmon4 and maroon.

Supplementary Table S1 Primers used for RT-qPCR analysis.

Supplementary Table S2 Detail information of sequencing reads from different sample groups.

Supplementary Table S3 Gene number of each module.

Supplementary Table S4 Connectivity of genes in salmon4.

Supplementary Table S5 Connectivity of genes in maroon.

## Abbreviations

ACO1: Aminocyclopropane-1-carboxylate oxidase
*A. flavus*: *Aspergillus flavus* L.
AP: Aflatoxin production
BAK1: Brassinosteroid insensitive 1-associated kinase 1
CDPKs: calcium-dependent protein kinases
CEBiP: chitin elicitor-binding protein
CERK: Chitin elicitor receptor kinase
DEGs: Differentially expressed genes
EFR: EF-Tu receptor
ETI: Effector-triggered immunity
FLS2: Fagellin sensitive 2
GO: Gene Ontology
HPLC: High performance liquid chromatography
II: Infection index
IVSC: *In vitro* seed colonization
KEGG: Kyoto Encyclopedia of Genes and Genomes
MAPK: Mitogen-activated protein kinase
PAMPs: pathogen-associated molecular patterns
PCA: principal component analyses
PRs: Pathogenesis-related proteins
PRRs: Pattern recognition receptors
PTI: pattern-triggered immunity
QTLs: Quantitative trait locus
*R*: Resistant
RLCK: receptor-like cytoplasmic kinases
RLK: receptor-like kinases
RT-qPCR: Real-time quantitative polymerase chain reaction
*S*: Susceptible
SNPs: Single nucleotide polymorphisms
STK: Serine/threonine kinase
SYP121: Syntaxin of plants 121
TF: Transcription factor
WGCNA: Weighted gene co-expression network analysis.
