# Supplementary figures and images for: Gene Co-expression Network Analysis of the Comparative Transcriptome Identifies Hub Genes Associated With Resistance to Aspergillus flavus L. in Cultivated Peanut (Arachis hypogaea L.)
Source: Front Plant Sci. 2022 Jun 15;13:899177. doi: 10.3389/fpls.2022.899177 (PMC9264616; doi:10.3389/fpls.2022.899177)

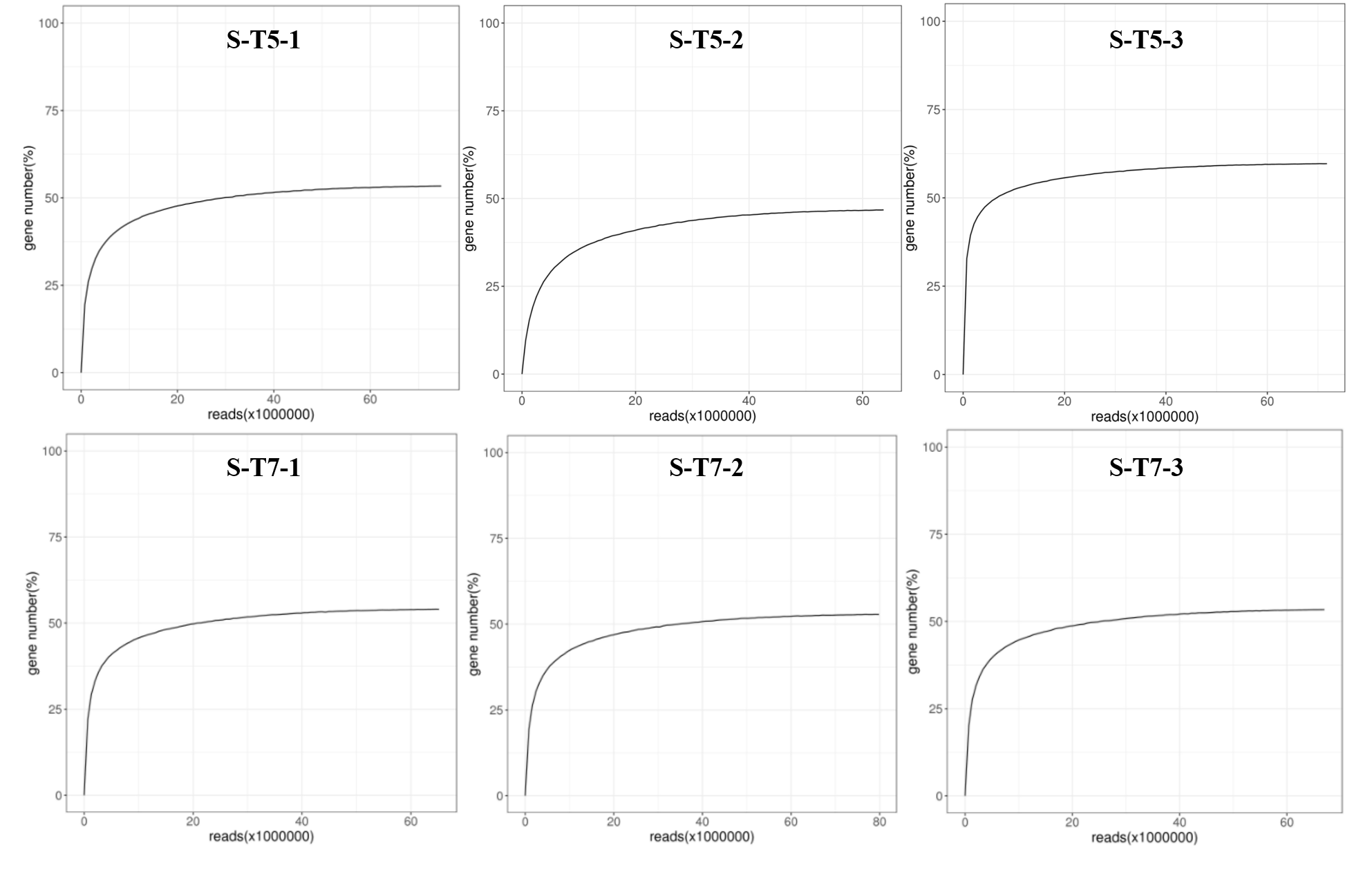

Supplement: Supplementary Figure S1 — Distribution of sequencing saturation of S-T5 and S-T7. [file Image_1.TIF]

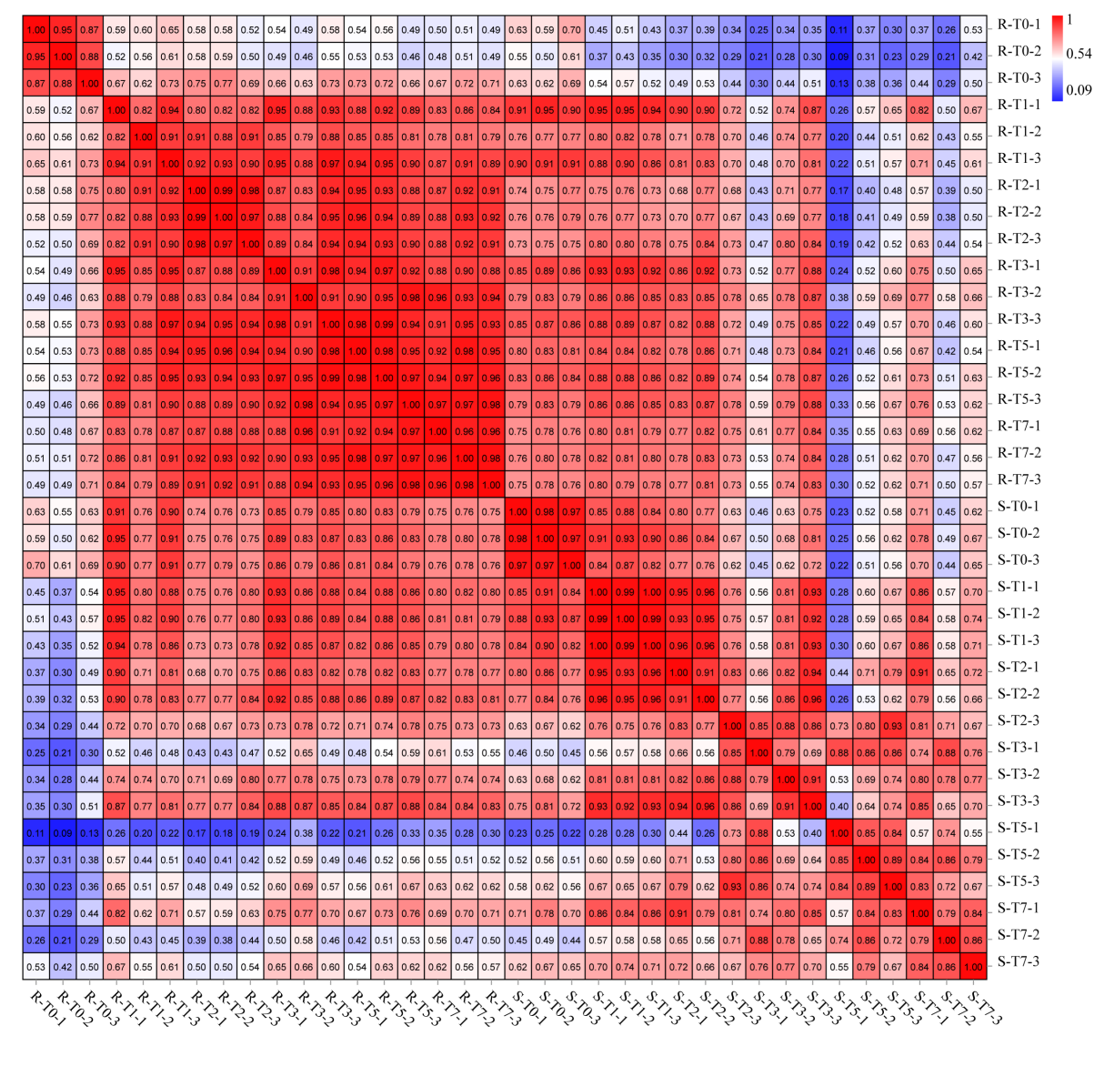

Supplement: Supplementary Figure S2 — Pairwise Pearson's correlation coefficients of the sequencing data of 36 samples. [file Image_2.TIF]

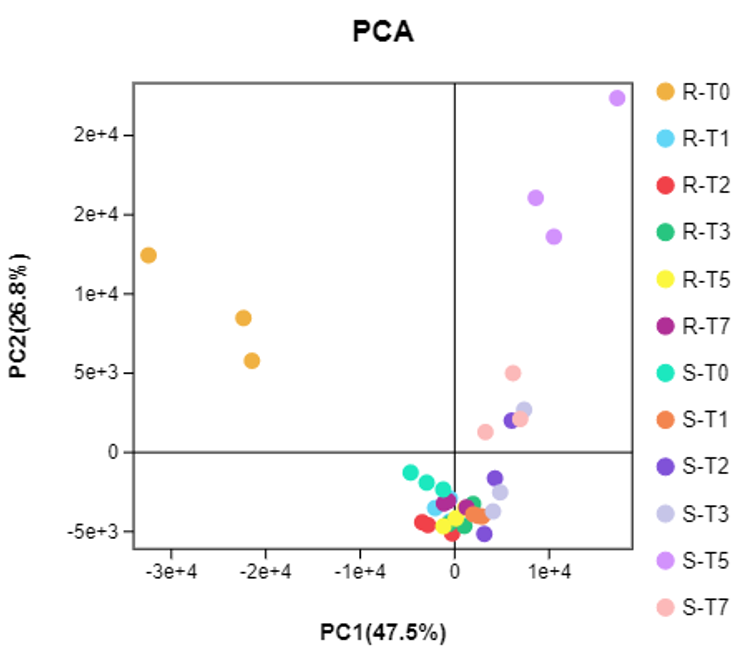

Supplement: Supplementary Figure S3 — Principal component analysis of the sequencing data of 36 samples. [file Image_3.TIF]

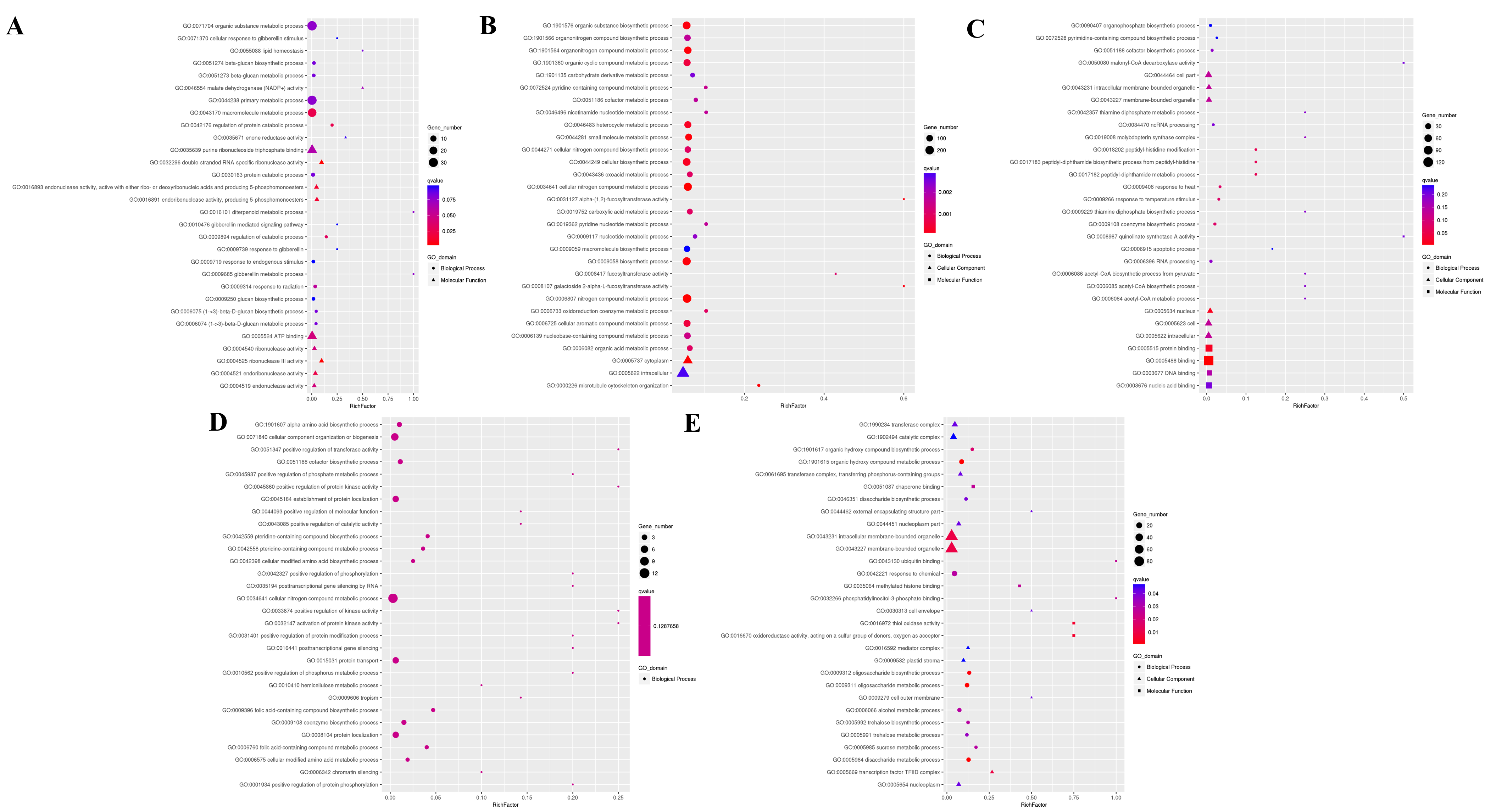

Supplement: Supplementary Figure S4 — Top 30 GO term enriched functional categories of DEGs upregulated uniquely in R at T1 (A), T2 (B), T3 (C), T5 (D), and T7 (E). [file Image_4.TIF]

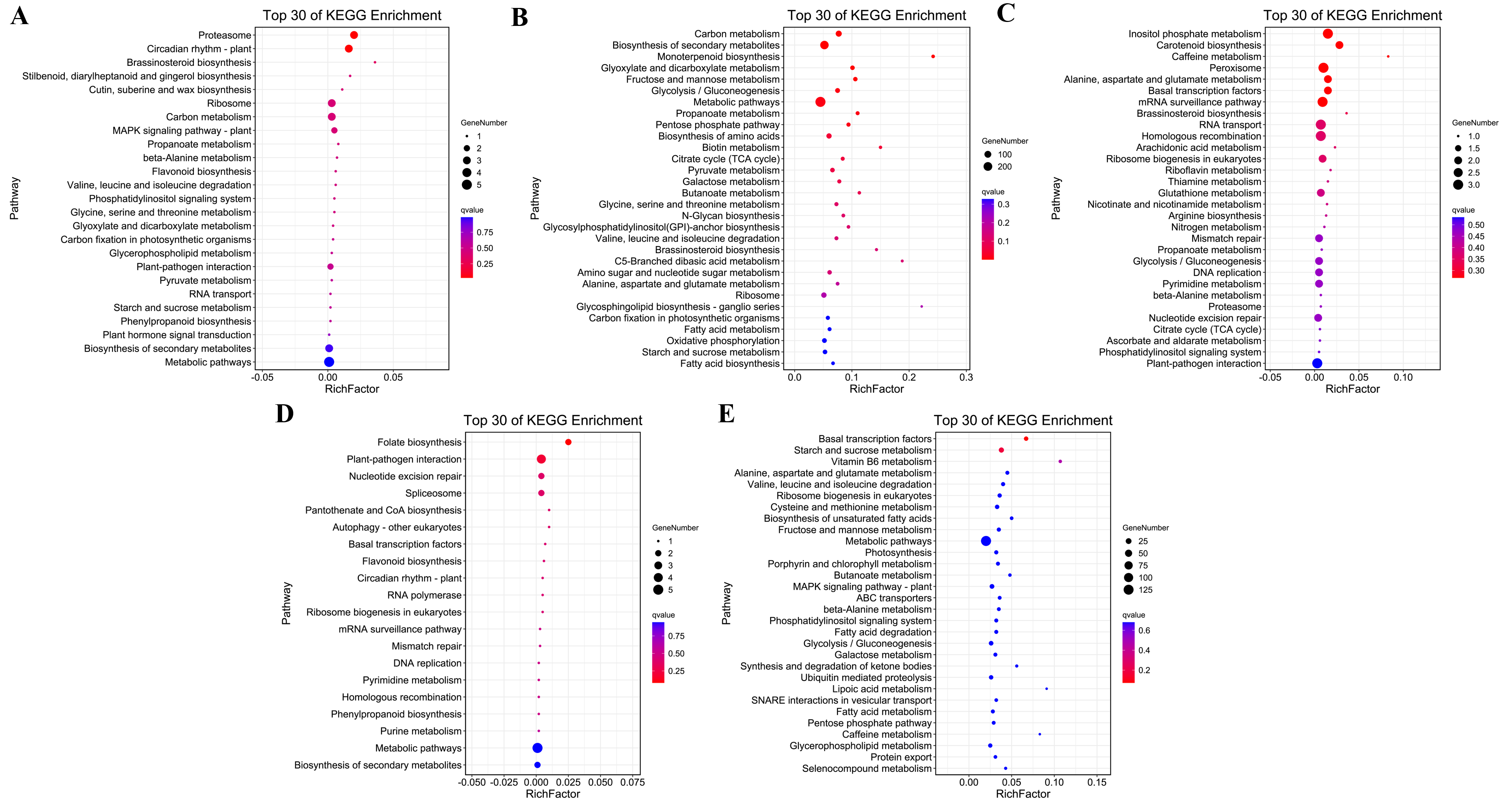

Supplement: Supplementary Figure S5 — Top 30 of KEGG enrichment of DEGs upregulated uniquely in R at T1 (A), T2 (B), T3 (C), T5 (D), and T7 (E). [file Image_5.TIF]

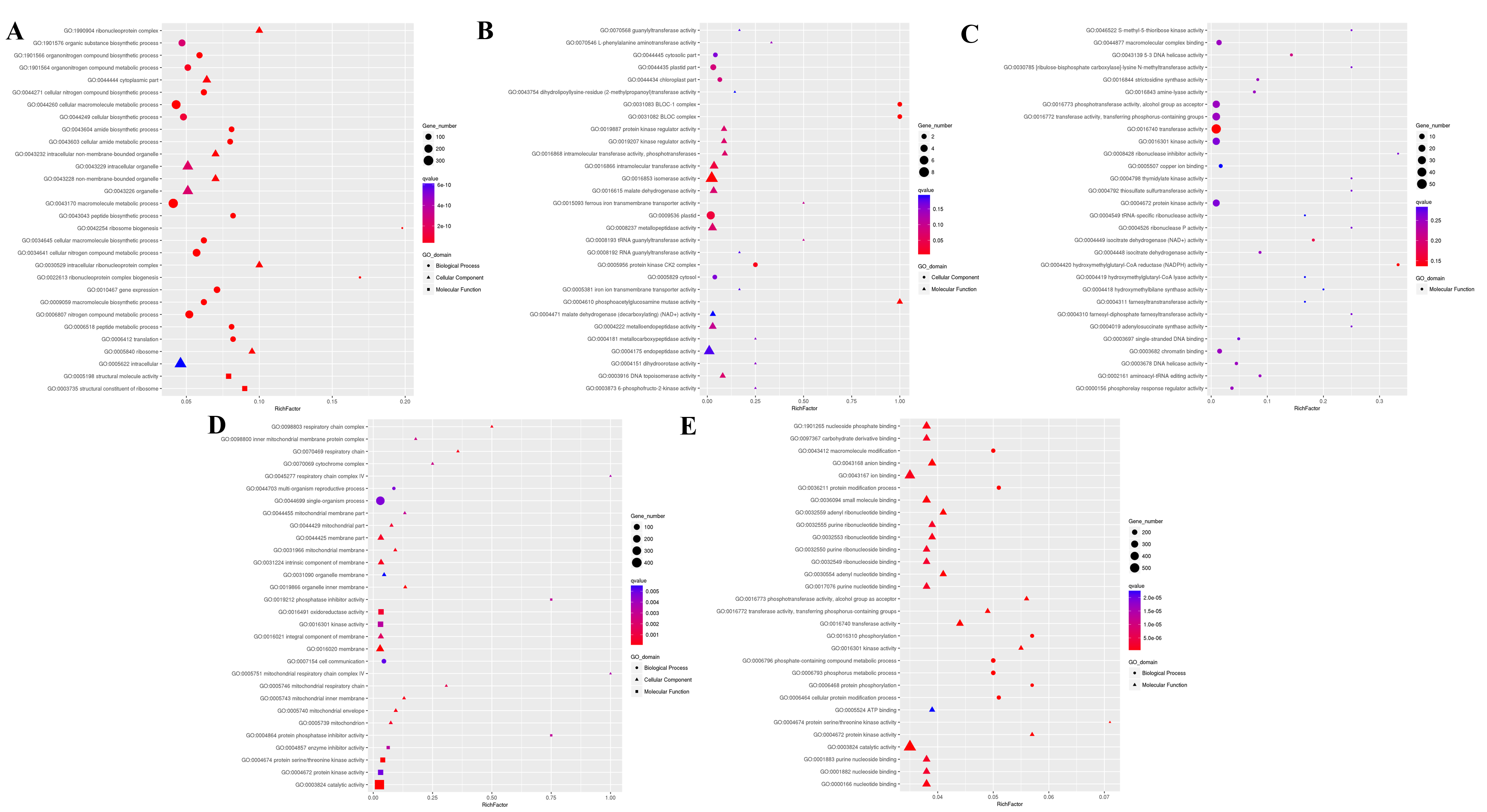

Supplement: Supplementary Figure S6 — Top 30 GO term enriched functional categories of DEGs upregulated uniquely in S at T1 (A), T2 (B), T3 (C), T5 (D), and T7 (E). [file Image_6.TIF]

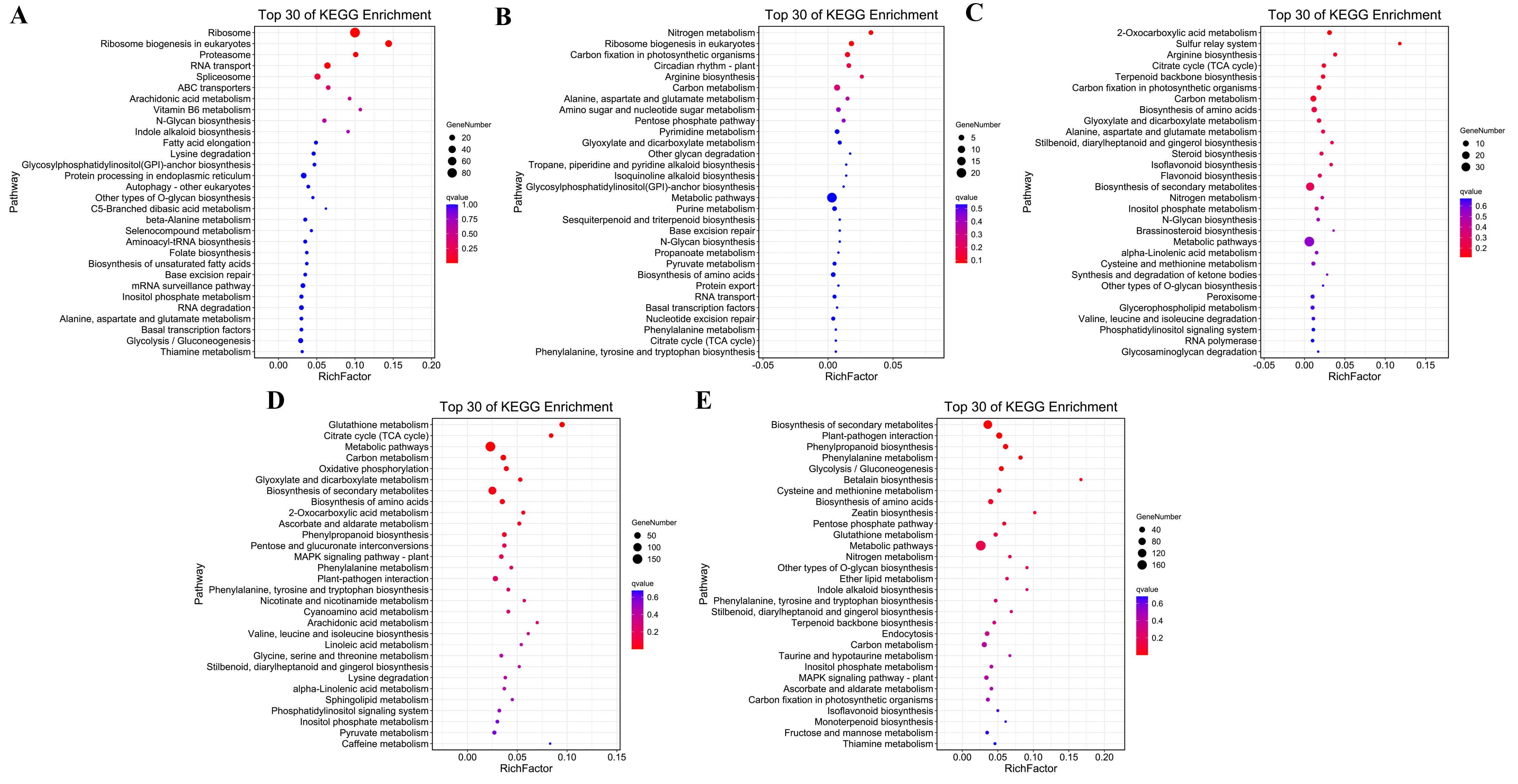

Supplement: Supplementary Figure S7 — Top 30 of KEGG enrichment of DEGs up-regulated uniquely in S at T1 (A), T2 (B), T3 (C), T5 (D), and T7 (E). [file Image_7.TIF]

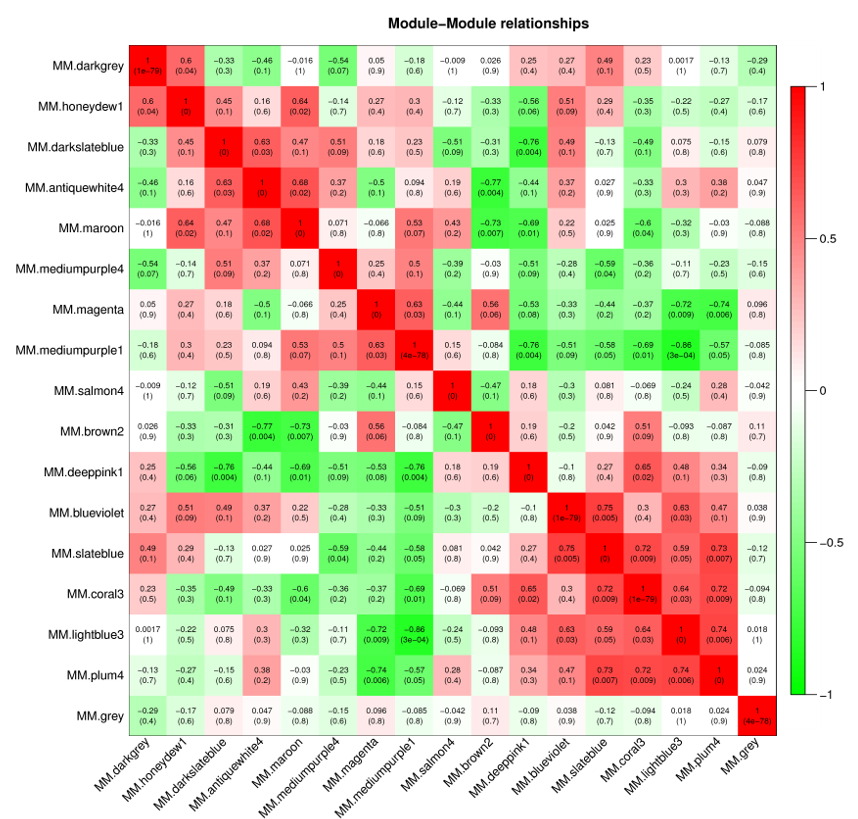

Supplement: Supplementary Figure S8 — Heat map of module-module relationship. [file Image_8.TIF]

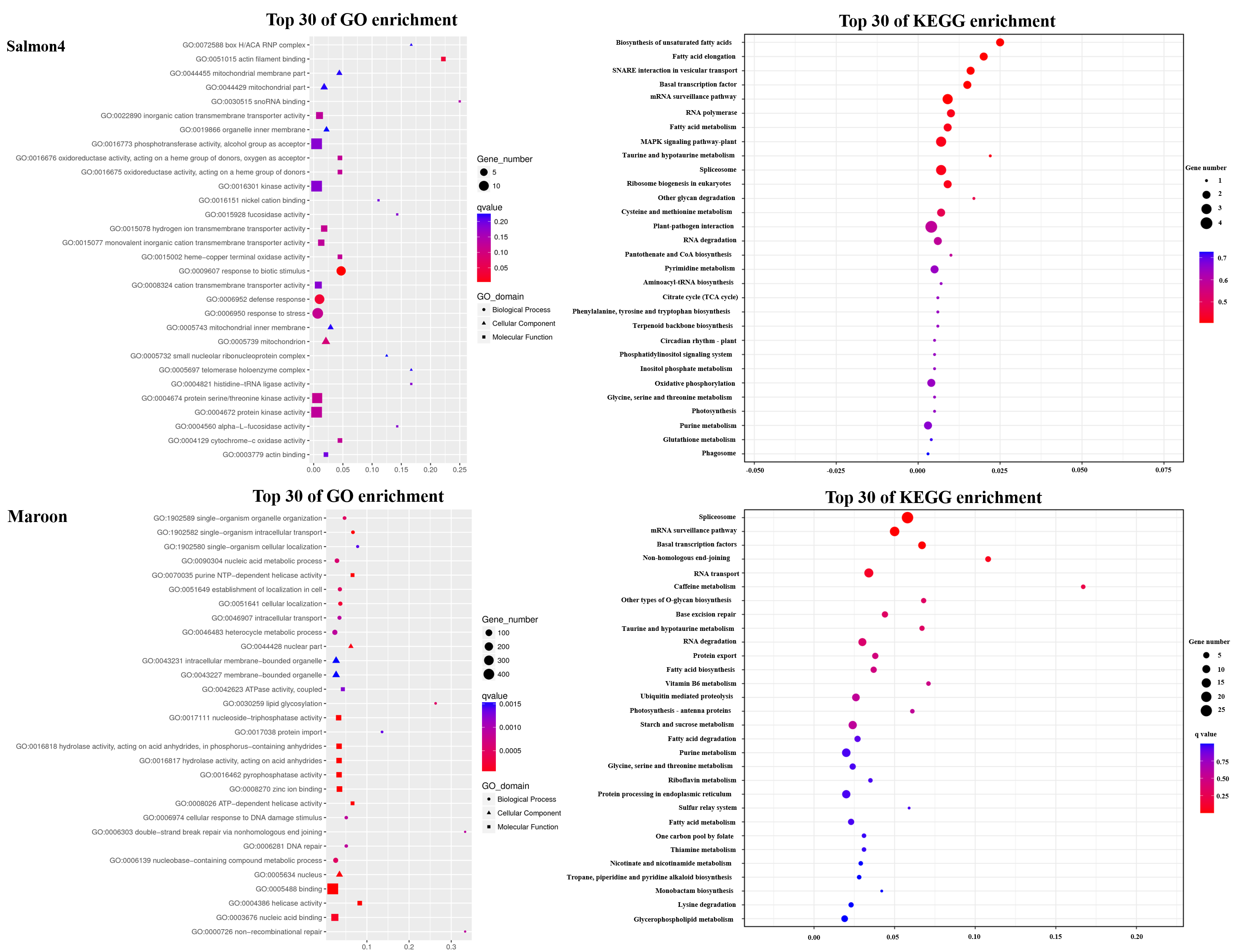

Supplement: Supplementary Figure S9 — Top 30 GO term enriched functional categories and top 30 KEGG enrichments of DEGs in salmon4 and maroon. [file Image_9.TIF]
